# Adjustment of Mechanical Properties of Medium Manganese Steel Produced by Laser Powder Bed Fusion with a Subsequent Heat Treatment

**DOI:** 10.3390/ma14113081

**Published:** 2021-06-04

**Authors:** Lena Heemann, Farhad Mostaghimi, Bernd Schob, Frank Schubert, Lothar Kroll, Volker Uhlenwinkel, Matthias Steinbacher, Anastasiya Toenjes, Axel von Hehl

**Affiliations:** 1 Leibniz Institute for Materials Engineering—IWT, Badgasteiner Str. 3, 28359 Bremen, Germany; f.mostaghimi@iwt.uni-bremen.de (F.M.); uhl@iwt.uni-bremen.de (V.U.); steinbacher@iwt-bremen.de (M.S.); toenjes@iwt-bremen.de (A.T.); axel.vhehl@uni-siegen.de (A.v.H.); 2 Department of Lightweight Structures and Polymer Technology, Technical University of Chemnitz, Reichenhainer Str. 31/33, 09126 Chemnitz, Germany; Bernd.Schob@mb.tu-chemnitz.de (B.S.); frschu@hrz.tu-chemnitz.de (F.S.); lothar.kroll@mb.tu-chemnitz.de (L.K.); 3 Faculty of Production Engineering, University of Bremen, Bibliothekstr. 1, 28359 Bremen, Germany; 4 Material Science and Testing, University of Siegen, Adolf-Reichwein-Straße 2, 57076 Siegen, Germany

**Keywords:** additive manufacturing, LPBF, TRIP, retained austenite, medium manganese steel, intercritical annealing, gas atomization

## Abstract

Medium manganese steels can exhibit both high strength and ductility due to transformation-induced plasticity (TRIP), caused by metastable retained austenite, which in turn can be adjusted by intercritical annealing. This study addresses the laser additive processability and mechanical properties of the third-generation advanced high strength steels (AHSS) on the basis of medium manganese steel using Laser Powder Bed Fusion (LPBF). For the investigations, an alloy with a manganese concentration of 5 wt.% was gas atomized and processed by LPBF. Intercritical annealing was subsequently performed at different temperatures (630 and 770 °C) and three annealing times (3, 10 and 60 min) to adjust the stability of the retained austenite. Higher annealing temperatures lead to lower yield strength but an increase in tensile strength due to a stronger work-hardening. The maximum elongation at fracture was approximately in the middle of the examined temperature field. The microstructure and properties of the alloy were further investigated by scanning electron microscopy (SEM), hardness measurements, X-ray diffraction (XRD), electron backscatter diffraction (EBSD) and element mapping.

## 1. Introduction

Additive manufacturing (AM) of metallic materials is becoming more and more established in a variety of industries due to its unrivaled low design constraints resulting from the tool-free layer-by-layer fabrication directly from CAD (computer-aided design) data. The high design freedom enables the creation of complex and individualized parts leading to a further intensification of super-lightweight construction. Among the different AM techniques, LPBF is considered one of the most promising ones as the process has proven to be capable of reliably manufacturing metallic parts with similar mechanical properties as conventionally produced parts such as casted or wrought material [1,2,3,4,5]. Additionally, the excess powder from the process can be reused, which reduces waste and, in turn, increases economic efficiency [6]. Although LPBF is suitable for a wide range of metals predominantly, Fe, Ni, Al, Mg, Ti and CoCr, the selection of commercially available materials is still limited [7].

From a material’s point of view, two main strategies have been applied to develop new powder alloys for AM. On the one hand, unique process conditions, such as the rapid solidification kinetics, have been utilized to fabricate or improve materials that are typically not producible by conventional production routes [8,9]. On the other hand, established alloys actually developed and optimized for conventional techniques have been processed and applied in AM [10,11,12]. Although the second approach barely takes advantage of the full potential of AM, materials can be processed that are otherwise difficult to manufacture, especially in large-scale production. For example, during LPBF, the high-energy laser beam moves fast across the powder bed, creating a melting pool that is at any time much smaller than the final part size [13]. Thus, materials that tend to macrosegregate can be fabricated more easily. One material class that tends to macrosegregate [14,15] but offers a unique opportunity for the development of cost-effective and light-weight parts with enhanced crashworthiness [16] are advanced high strength steels [14,15,16,17].

AHSS are commonly classified into three generations. Dual phase (DP), complex phase (CP) and transformation-induced plasticity (TRIP) steels, which belong to the first-generation AHSS, are widely used today as sheet metal materials. They exhibit solid strength and ductility properties and, in case of the TRIP steels, additionally, a safety buffer by strain hardening in the case of a crash [17]. Despite their good mechanical properties, they have been hardly investigated in AM [18,19,20].

The second-generation AHSS is the twinning-induced plasticity (TWIP) steels. These steels consist of high amounts of Mn with 15–30 wt.% as well as additional alloying elements predominantly Al, C and Si [21]. These high manganese steels show superior mechanical properties through the plasticity-enhancing mechanism, but their high manganese and aluminum contents cause a particularly strong tendency to macrosegregate. Hence, they are difficult to produce on a large scale, i.e., by strip casting. This fact, together with the fully austenitic microstructure, makes high manganese steels a good candidate for AM. Previous studies have shown that high manganese steel can be processed by LPBF and can achieve similar mechanical properties as the sheet material of the same alloy, even in a not post-treated condition. The samples showed high strength and good ductility due to deformation twins [13]. The solidification kinetics of the LPBF-process led to a larger grain size compared to recrystallized samples and, therefore, to a decrease in austenite stability, which is also caused by a loss of manganese during the process [13]. In addition, it reduced elemental segregation compared to ingot- and strip-cast material, which is normally the reason for a post-treatment to enhance mechanical properties, and resulted in the formation of small areas of martensite in the as-built state in a typically pure austenitic microstructure [5,7]. This can be contributed to partial compensation of the internal stresses and dislocations by the LPBF-process and led to higher strength compared to conventionally produced steel [5]. The minor residual porosity and impurities resulted in a lower elongation. However, the LPBF-samples showed equal work hardening capacities, despite martensite in the as-built-state, and linear increase in true stress, therefore the same deformation characteristics, i.e., TWIP and TRIP-effect, though with a strong anisotropy typical for additive manufacturing [5]. TWIP and TRIP have also been found in the LPBF-as-built-state of other alloys, such as martensitic stainless steel [22] or high strength tool steels [18], with both showing higher austenite contents after processing by LPBF than expected. While the studies show outstanding mechanical properties and suppression of macrosegregation, the fully austenitic structure leads to crack initiation during spot welding, which decreases the cost-effectiveness and applicability in many industries, such as the automotive industry.

To overcome this bottleneck, medium manganese steels, which belong to the third-generation AHSS, are under consideration. They have shown a superior combination of strength and ductility, which can be placed between the values of the first and second-generation AHSS. Medium manganese steels provide an ultra-fine grained austenitic-ferritic/martensitic microstructure, and its ductility is caused by the activation of the plasticity-enhancing strain-induced transformation mechanism (TRIP-effect) or by a combination of both TRIP and TWIP. Whether TRIP and/or TWIP is activated or suppressed in an alloy during plastic deformation strongly depends on their stacking fault energy (SFE). This parameter controls the dissociation distance of partial dislocations, and its effect on the aforementioned transformation-induced mechanisms is well known for face-centered cubic (fcc) metals. Low SFE values <18 mJ·m^−2^ have been reported to activate TRIP, while values in the range of 12–35 mJ·m^−2^ coincide with the occurrence of TWIP [23]. However, the mentioned value ranges vary slightly in the academic discourse [24,25]. Nevertheless, since both value ranges overlap each other, both mechanisms can be activated simultaneously by tailoring the SFE by the chemical composition. The dependency of the elements Mn, Al, Si and C on the SFE can be found here [26]. Besides the chemical composition, the SFE depends on the microsegregation (Suzuki effect) temperature and the grain size [27]. Since medium manganese steels exhibit a complex microstructure, these aforementioned effects can be controlled by the amount and the stability of metastable retained austenite (RA), which can be adjusted via a heat treatment process of intercritical annealing [28,29,30,31,32]. In addition, austenite stability can be controlled by certain elements, manganese and carbon being the most relevant. During intercritical annealing, these elements partition from ferrite or martensite into austenite, stabilizing it chemically [33]. Further common alloying elements for controlling SFE are Si and Al [29]. Moreover, Al increases the temperature range for intercritical annealing to higher temperatures [34]. This allows the annealing time to be reduced by enhancing the Mn diffusion without decreasing the stability of the retained austenite otherwise detectable at higher annealing temperatures [31,35,36].

Despite excessive research on this material as sheet metal, the feasibility of LPBF-processing of medium manganese steel, the third generation of AHSS, with a manganese content between 3 and 12 wt.% [37], has hardly been studied so far. The results of Höfemann et al. and Zapf et al. originate from the same research project as this study and show the weldability of successfully LPBF-processed medium manganese steel [38,39], albeit on a slightly different alloy than the one presented here. The following study investigates the applicability of medium manganese steel with low Mn content for additive manufacturing. Therefore, the material was first gas atomized, fabricated by LPBF and heat-treated to determine the mechanical properties and microstructure formation.

## 2. Materials and Methods

### 2.1. Atomization

The composition of the medium manganese pre-alloy chosen for this study is FeMn5Al2Si0.5C0.2. The powder material was produced in a powder production plant described in [40]. Pure iron, manganese, silicon and aluminum were melted in an Al_2_O_3_ crucible under a protective argon atmosphere to a melt temperature of 1715 °C (corresponds to about 200 °C above liquidus temperature). After a homogenization time of 15 min, the melt was atomized in a Close-Coupled-Atomizer (INDUTHERM Erwärmungsanlagen GmbH, Walzbachtal, Germany) equipped with a convergent-divergent gas nozzle [41]. The process parameters used for the atomization are summarized in Table 1. The powders collected at the bottom of the plant were sieved and air classified to a size fraction of 20–63 µm. Afterwards, the particle size distribution was measured by laser diffraction analysis (Malvern Mastersizer 2000, Malvern Panalytical GmbH, Kassel, Germany). The particle shape was quantitatively and qualitatively determined by static image analysis (G3, Malvern Panalytical, Malvern, UK) and scanning electron microscope (SEM) (VEGA II XLH, TESCAN, Brno, Czech). The flowability was measured according to DIN EN ISO 4490, and the bulk density was measured according to DIN EN ISO 3923-1.

The chemical composition of the powder as well as of the processed samples were investigated by atomic absorption spectrometry (for Mn and Al), gravimetry (for Si) and infrared absorption (for C).

### 2.2. Stacking Fault Energy

The stacking fault energy was calculated by a subregular solution thermodynamic model originally proposed by Olson and Cohen [42] and previously modified and applied by Zambrano for Fe–Mn–Al–Si–C alloys [26]. The model uses the approach by Yang and Wan [43] to calculate the molar thermochemical Gibbs free energy $∆G_{chem}^{\gamma \to \varepsilon }$ from the change of Gibbs free energy Δ*Gγ*→*ε* for each element upon the phase transformation of austenite to martensite. The approach also accounts for the interactions between the components via interaction parameters *Ωγ*→*ε*. The magnetic transition energy $∆G_{mag}^{\gamma \to \varepsilon }$ due to Néel transition is taken into account by the Inden-Hilert-Jarl model [44] with an equation for the magnetic moment proposed by Jin et al. [45]. The influence of the grain size is also considered by an excess term for the Gibbs energy $∆G_{ex}^{\gamma \to \varepsilon }$. The equations for the described models, as well as more detailed information about them can be taken from [26]. With the calculated free energies, the SFE can be expressed by Equation (1) [42].

(1) $$SFE=2\rho \left(∆G_{chem}^{\gamma \to \varepsilon }+∆G_{mag}^{\gamma \to \varepsilon }+∆G_{ex}^{\gamma \to \varepsilon }\right)+2\sigma^{\gamma /\varepsilon }$$

Here, ρ is the molar surface density, and $\sigma^{\gamma /\varepsilon }$ is the interfacial energy between the phases γ and ε. The parameters used for this model are listed in Appendix A. With the before-mentioned alloy composition, the resulting SFE value is 21 mJ × m^−2^ and thus in the favored range.

### 2.3. Laser Powder Bed Fusion Process

The development of the laser parameters for processing the manganese steel alloy was carried out on an SLM 125^HL^ equipped with a 400 W IPG fiber laser (SLM Solutions, Lübeck, Germany). The selected layer thickness of 60 µm and a laser spot diameter of 100 µm aiming at a high buildup rate. The platform temperature was set to 200 °C.

Based on single track investigations and cubic test specimens with the edge length of 5 mm, the scan speed, hatch distance and laser power were varied and evaluated in a range of volume energy inputs (*E_V_*) between 48 J/mm^3^ and 146 J/mm^3^. *E_V_* is calculated from laser power *P_L_*, layer thickness *D_S_*, scan speed vs. and the hatch distance Δ*y_S_* according to Equation (2). The applied scanning strategy corresponds to a bidirectional laser motion with a slice-by-slice rotation of 67°.

(2) $$E_{V}=\frac{P_{L}}{D_{S}*v_{S}*\Delta y_{S}}$$

The samples were built on block supports with a wall thickness of 0.5 mm. Subsequently, these specimens were separated from the substrate and freed from any support and adhesions to determine the relative density. Afterwards, these specimens were examined by means of microscopic micrographs on a Keyence VHX 600 (Osaka, Japan) laser microscope. The evaluation of the micrographs resulted in the highest achievable relative density up to 99.9% within this parameter investigation. The energy input of 111.1 J/mm^3^ achieves the highest relative density. Based on these investigations, the process parameters could be narrowed down to a scanning speed of 500 mm/s at a laser power of 300 W and a hatch distance of 90 µm. A higher energy input leads to increased spatter formation and defects, such as bonding defects and pores. A lower energy input led to bonding defects due to too low melting power. Following the density determination, cylindrical samples were manufactured for the investigations of the mechanical properties (according to DIN 50125-B4). In view of the amount of powder available, only upright specimens were produced.

### 2.4. Heat Treatment and Mechanical Testing

In order to raise the ductility by increasing the retained austenite content, the samples have to undergo an intercritical annealing process. The selection of a suitable annealing temperature and annealing time is of decisive importance for adjusting the stability of austenite. A temperature range was estimated by calculations using Thermo-Calc Software (Stockholm, Sweden) TCFE Steels/Fe-alloys database version 11. According to these calculations, Ac1- and Ac3-temperature are at 650 and 945 °C. The annealing temperatures were oriented close to and one just below Ac1 in order to still benefit from the fine-grained as-built microstructure. Finally, the heat treatment was investigated at 8 discrete temperature steps, between 630 and 770 °C, while 3 different annealing times (3, 10 and 60 min) were applied for every examined temperature step.

The heat treatment was performed using a chamber furnace under an air atmosphere. Because of the surface reaction and decarburization tendency in the air atmosphere, the samples were manufactured with stock material, being removed after heat treatment. The cylindrical samples were placed into the hot furnace, and the annealing time was measured as soon as the annealing temperature was reached in the core of the samples. To determine the core temperature, an additional steel body with the same size as the cylindrical specimens equipped with a mantel thermocouple Type K that was placed in a bore in its core was also placed in the chamber. The total time until the core reached the annealing temperature was between 13 and 15 min, depending on the target temperature. After the completion of the annealing time, the specimen was quenched in oil at room temperature without forced agitation.

From the cylindrical sample tensile tests, the specimens were machined by turning according to DIN 50,125 type B with a test diameter of 4 mm. The tensile tests were performed according to DIN EN ISO 6892-1. In total, 3 samples were tested for each combination of heat treatment parameters, and 5 samples in the as-built condition.

### 2.5. Microstructure Characterization

For the microstructural characterization, longitudinal cross-sections of the used tensile specimens were prepared by wire cutting. Prior to SEM (Vega II XLH, TESCAN, Brno, Czech), the specimen w etched with 3% alc. HNO_3_ and sputtered with gold. Hardness curves along the longitudinal axis were determined with the HV1 measurement method.

The EBSD samples were mechanically polished, using a 1-µm diamond lubricant for 10 min and finely polished with OP-S suspension and distilled water for 30 min. Measurements were conducted on a Helios G4 FEG-SEM (Thermo Fisher Scientific, Waltham, MA, USA) equipped with an EBSD Hikari Plus camera (Ametek EDAX, Berwyn, PA, USA). An acceleration voltage of 20 kV, a beam current of 6.4 nA and a step size of 0.03 µm were used to separate austenite and ferrite (martensite) of the fine grain structure in a small area of 20 × 20 µm^2^. 

For X-Ray diffraction measurements, the samples were etched at about 100 µm with 80% phosphoric and 20% sulfuric acid. Measurements were carried out on an ETA 3003 diffractometer (XRD Eigenmann GmbH, Schnaittach-Hormersdorf, Germany) using Cr-k(alpha) radiation and a 0.5 mm primary X-ray beam. The phase analysis was performed over a range from 60–164° with a step size of 0.05° and a collecting time of 800 s and evaluated using the Rietveld method implemented in TOPAS 4.2 (Bruker-AXS, Billerica, MA, USA).

## 3. Results

### 3.1. Powder Characterization

**Figure 1:**
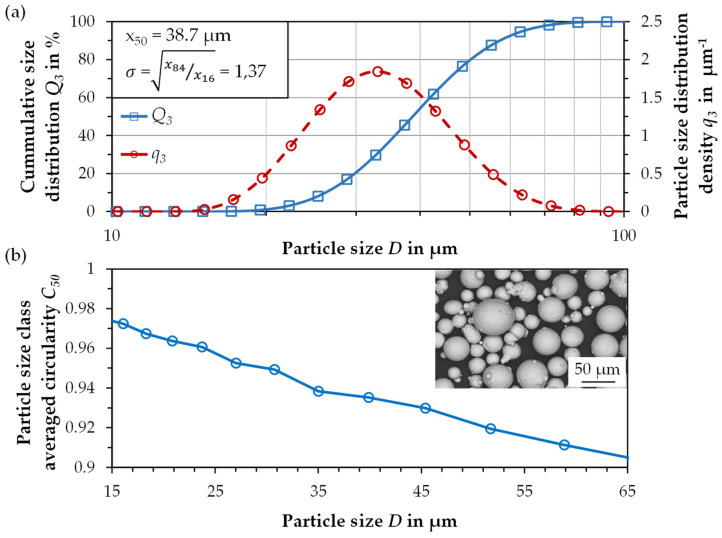
(**a**) The particle size distribution and (**b**) particle size class averaged circularity.

The results of the particle characterization of the FeMnAlSiC powder in the size range of 20–63 µm are summarized in Figure 1a. The resulted mean particle diameter d_50,3_ yields to 38.7 µm with a geometrical standard deviation σ = (x_84_/x_16_)^0.5^ of 1.37, indicating a narrow size distribution. The bulk density is 4.25 g cm^−3^. The resulting flowability according to the Hall Flowmeter test is 17.1 s (50 g)^−1^. Both the density and flowability values are in a comparable order of magnitude to other steel powders from the literature [46,47]. The morphology of the particles was determined qualitatively by SEM and quantitatively by static image analysis. For the latter, the particle class averaged circularity C_50,_ one of the most widely used form factors, was calculated from the image data to quantify the particle shape. Here, the circularity is defined as the circumference of a circle with the same projected area as the original particle divided by the particle’s circumference. As shown in Figure 1b, the atomized particles are mostly spherical shaped and reach C_50_-values above 0.9. Nevertheless, most particles still show few attached satellites, which can decrease the powder density and the flowability [48]. However, since the powders show sufficient high flowability and density values and the spreadability during LPBF was satisfactory, no flowability enhancement is required.

The chemical analysis shows that 0.4 wt.% of the manganese content burns off during melting and atomization due to the high manganese vapor pressure (Table 2). The LPBF process burned off a further 0.64 wt.% Mn, so that just under 4 wt.% of the set 5 wt.% Mn remained. This must be taken into account in the alloy development.

The change of the alloy composition also slightly affects the SFE value, which is nevertheless still in the favored range with 24 mJ × m^−2^.

### 3.2. LPBF

A robust process window for the LPBF process could be developed within the investigations, which has a very low susceptibility to faults with regard to the sensitivity to the scan speed, if the tolerance of +/−5% is maintained.

The LPBF process is characterized by a certain amount of smoke and spatter emission, which influences the absorption of the laser energy and the melting behavior. Furthermore, an already mentioned manganese burn-off after the LPBF process of about 15 wt.% to the previous Mn content was detected. As a result of these processes, pores are formed to a certain extent, which was detected by means of microscopic micrographs showing a relative density up to 99.9%. The remaining defects in the samples can, in all probability, be significantly reduced if a reduced layer thickness is applied. These results, in conjunction with the overall process observations, show that the material is fundamentally suitable for the LPBF process and represents a relevant and high potential in the medium term based on further iteration steps.

### 3.3. Mechanical Properties

In the as-built condition, the material has a high yield strength of 844 ± 49 MPa, a tensile strength of 952 ± 71 MPa, with a low elongation at fracture of 5.3 ± 4.3%. In particular, the high standard deviation of the elongation at fracture (values from 0.4 up to 11%) is only to a limited extent caused by pores but rather because of small bonding defects between the layers. These bonding defects emerge because of the formation of spatter during laser exposure, which settled down on the build platform, also on the specimens before re-exposure, which in some cases led to incomplete layer bonding, and requires further process adjustments.

**Figure 2:**
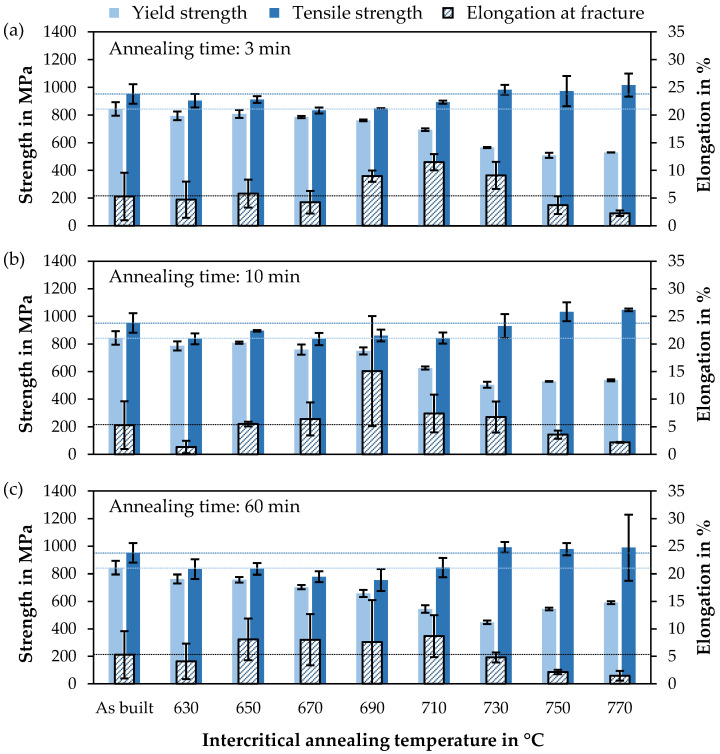
Mechanical properties as a function of annealing temperature for 3 annealing times ((**a**) 3 min, (**b**) 10 min and (**c**) 60 min) with the results as-built as horizontal lines.

**Figure 3:**
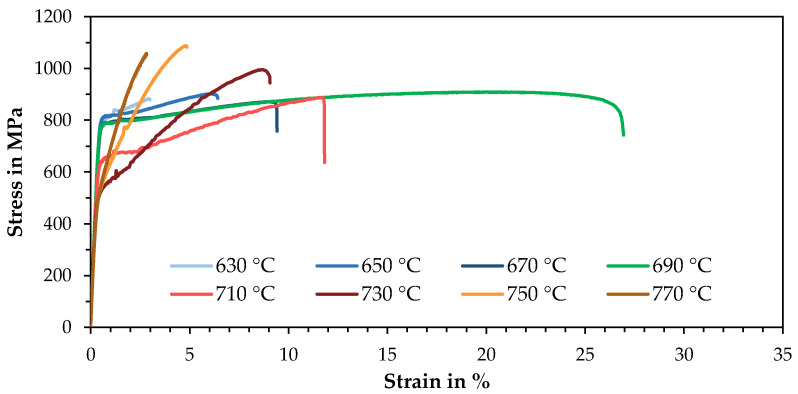
A stress-strain diagram of LPBF specimens after an intercritical annealing process of 10 min at different temperatures.

With increasing annealing temperature and time, the yield strength is reduced (Figure 2 and Figure 3) due to the recovery of retained austenite, grain coarsening, and tempered martensite. The lowest investigated annealing time of 3 min already shows a clear effect on the mechanical properties, e.g., reducing the yield strength by 50 MPa at the lowest tested temperature of 630 °C. Above a temperature of 750 °C, however, the yield strength raises again with increasing temperature and annealing time. This may be due to the rapid transformation of the austenite formed, which occurs even before the offset yield point R_p0.2_ is reached.

The tensile strength is relatively constant, at about 50 to 100 MPa below the as-built state, in a temperature range up to 710 °C and then raises significantly from 730 °C, reaching over 1 GPa at 750 and 770 °C, due to high strain-hardening rates caused by TRIP during plastic deformation. The influence of the annealing time is less dominant for the tensile strength than for the yield strength.

The elongation at fracture shows a quite symmetrical curve with a maximum at 690 or 710 °C, depending on the annealing time. A longer annealing time causes an enhanced elongation at fracture at low temperatures and a decrease at higher temperatures. This is directly related to the austenite stability. If the annealing time is quite short or the temperature low, only a small volume of austenite with high stability is present. If the temperature or time is increased, whereby the effect of the temperature appears to be greater, more austenite is formed in the annealing process, which is, however, less stable. The highest elongation at fracture, based on the maximum value achieved, is at 710 °C for 3 min annealing time and at 690 °C for 10 and 60 min. Here, the austenite stability is optimally set so that it successively transforms deformation induced. If the temperature is too high or the annealing time too long, this results in a decrease in elongation at fracture, but with a simultaneous increase in tensile strength, since the majority of the austenite already turns into martensite at low deformation.

With an annealing time of 10 min and an annealing temperature of 690 °C, a maximum elongation at fracture of 26.5% was reached. Higher temperatures lead to an increasing work hardening rate, higher tensile strength at the expense of elongation at fracture and yield strength.

### 3.4. Hardness and XRD

**Figure 4:**
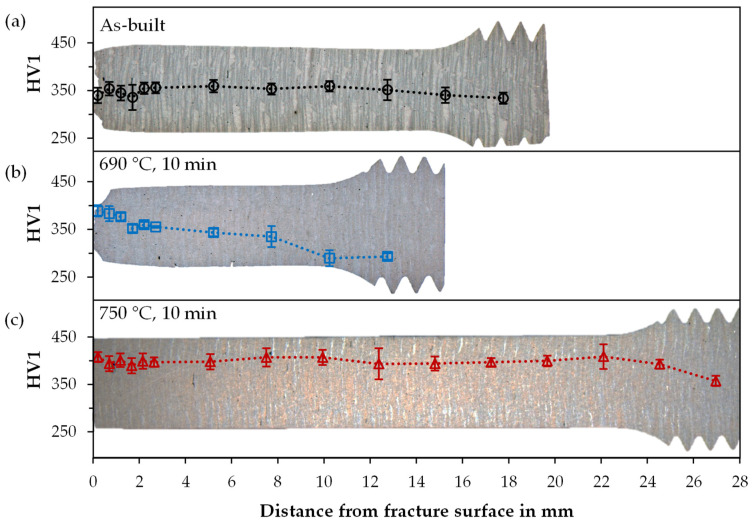
The hardness curves along the longitudinal axes of tested tensile samples (**a**) as-built and after intercritical annealing for 10 min at (**b**) 690 °C and at (**c**) 750 °C.

The varying austenite stability due to the applied heat treatment is also evident in the hardness curves. The longitudinal cross-section of a tensile specimen shows the progression from the fully loaded fracture edge to the barely loaded thread (Figure 4). Here, the as-built specimen shows an almost constant hardness curve of about 350 HV 1. The specimen heat-treated at 750 °C has the highest average hardness. It also shows a constant progression of about 400 HV 1 along the test length, but with a slight increase at the fracture edge (408 HV 1) and a drop in hardness to 357 HV 1 in the thread area. The specimen annealed at 690 °C for 10 min and exhibited the highest elongation at fracture also shows a clear hardening towards the fracture edge. The hardness in the thread is only 290 HV 1, which suggests a high austenite content. The value increases to 335 HV1 at the beginning of the test length and successively to 389 HV1 up to the fracture edge.

**Figure 5:**
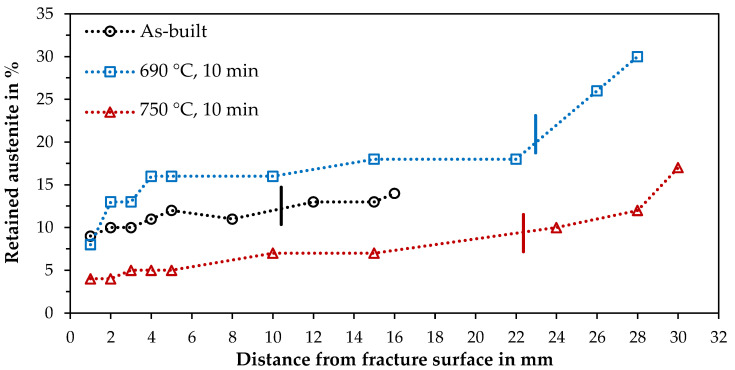
Retained austenite along the longitudinal axis of tested samples from the fracture surface to the thread as-built and intercritically annealed at 690 and 750 °C for 10 min. The dash marks the end of the test length (not the identical samples as for the hardness curves, therefore different lengths).

The results of the XRD measurements fit well with these results. The retained austenite content in the thread shows the lowest value for the as-built sample at 14%, which decreases only slightly to 9% towards the fracture edge (Figure 5), thus, indicating high stability of the retained austenite produced in the LPBF process. At 750 °C, the initial retained austenite content is 17%, which drops to 7% at the beginning of the test length and to 4% at the fracture edge. The 690 °C sample starts with the highest RA content of 30%, which then decreases from 18 to 8% along the test length, demonstrating the austenite transformation due to deformation (TRIP).

### 3.5. Microstructure and EBSD

**Figure 6:**
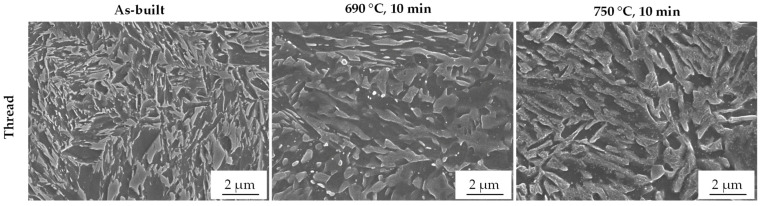
SEM images within the thread region as-built and after intercritical annealing at 690 °C and at 750 °C for 10 min.

The microstructure in the as-built state is mainly fine-grained and composed of a martensitic-bainitic matrix with fine dispersed retained austenite being at an effective size of less than 2 µm in diameter. The results of the heat-treated samples show that during intercritical annealing of the as-built samples, the austenite fraction increases and the retained austenite grows into larger islands through the intercritical annealing process (Figure 6). Additionally, it was seen that at an intercritical annealing temperature of 750 °C, a substructure in the RA islands indicates a martensitic transformation, and precipitates can be seen. Therefore, it is suggested for the 750 °C sample that a transformation to martensite already happened during cooling from intercritical annealing temperature, and precipitations are formed from martensite.

**Figure 7:**
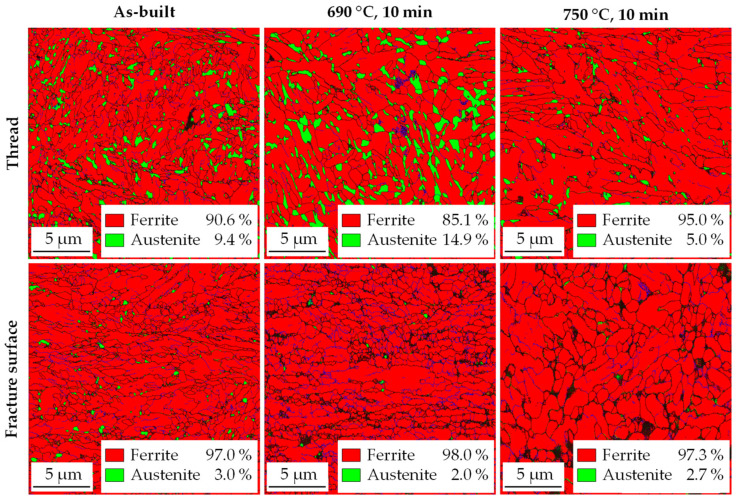
EBSD phase images of the threaded area and near the fracture surface as-built and after intercritical annealing at 690 °C and 750 °C for 10 min. The black areas could not be assigned.

While EBSD results also clearly show an increase in RA due to intercritical annealing at 690 °C as well as RA transformation close to the fracture surface (Figure 7), the levels of austenite content are generally lower compared to XRD measurements. The samples for EBSD were mechanically prepared, whilst the samples for XRD were electrolytic ablated by an etchant without any mechanical forces applied. In addition, the measuring area for high-resolution EBSD is significantly smaller, and thus, the statistics are worse than in the XRD measurement. Hence, the comparison of the measurement results allows conclusions to be drawn about the mechanical stability of the residual austenite. The difference in measurements is the smallest for the as-built sample (9.4–14% as the highest amount in the thread, measured by XRD (Figure 5)), a very fine dispersed distribution of retained austenite. The relatively highest deviation occurs for the 750 °C sample with 5.0% compared to 17%. The highest RA content of 14.9% is present at 690 °C. The transformation of the austenite at the fracture surface is observed in all three specimens, with a remaining content of 2.0–3.0%.

## 4. Summary and Discussion

Gas atomization and LPBF process of the resulting medium manganese steel FeMn4Al2Si0.5C0.2 could be carried out successfully. To achieve the required manganese content in parts, a super alloying addition of manganese is mandatory, as both processes cause a burn-off of manganese, here of 21.4% in total. Further investigations on influencing factors for the reduction of the burn-off need to be done. Aiming to upscale the LPBF process to larger components, the resulting greater amount of evaporations require effective gas flow management.

The development of the LPBF process led to a part density of 99.9%. However, the process was still characterized by melt spatter, which partially resulted in greater bonding errors between layers, causing high standard deviation in the mechanical properties, especially the elongation at fracture. The process, therefore, requires careful control and further adjustments of the process parameters, as well as the material. The contribution of silicon and aluminum on the process instability due to Si influencing the melt viscosity and surface tension must be further investigated in order to reduce these effects.

It could be shown by tensile tests that the best mechanical properties by the means of elongation and strength could be achieved using an intercritical annealing temperature of 690 °C and a time of 10 min.

During intercritical annealing, two major processes happen:

The transformation of ferrite into austenite depending on the temperature applied and partitioning (diffusion) of Mn and C into the austenite, stabilizing the austenite chemically. Besides phase transformation, the as-built microstructure is rather refined and has a high dislocation density affecting the first transformation into martensite during quenching (during LPBF).

A higher annealing temperature favors the formation of more austenite during annealing due to the partitioning of Mn and C into austenite. Additionally, with increasing annealing temperature and phase transformation into austenite, the effective amount of sub-micron defects such as dislocation cell structures is continuously reduced, resulting in a decreased hardness and yield strength, enhanced by grain coarsening and tempering the martensite.

With increasing distance to Ac1 the thermally induced ferrite to austenite transformation is resulting in an increased austenite fraction. Thus, the concentration of Mn and C in austenite decreases because the soluting volume is increased. As the volume of RA increases, the concentration of these elements in the austenite fraction is consequently lower, and the chemical stability against transformation into martensite is reduced. Additionally, large austenite fractions tend to transform more easily during elastic and plastic deformation than finely dispersed pinpoint austenite. For those two reasons, the phase stability of RA decreases, resulting in transformation during cooling and a, therefore, smaller fraction of RA in the final microstructure at RT. If the annealing temperature is optimally set, the amount of austenite after cooling is maximized at around 30 wt.% at intermediate phase stability, and therefore, strain-induced plasticity occurs over a wide strain range, reaching maximum elongation of up to 26.5%.

At annealing temperatures exceeding 700 °C, the RA volume achieved by the heat treatment at RT decreases because of the lack of chemical-induced thermal stability. During tensile testing, it could be derived that the metastability of the retained austenite is at lower mechanical stability because of the rather fast transformation during testing. The RA transformation then leads to significant strain hardening and, therefore, to higher tensile strengths.

The transformation and varying stability of RA is further shown by the comparison of two areas, thread and fracture surface, by hardness, XRD and EBSD measurements on samples at different annealing temperatures. Results indicate the highest RA stability in the as-built state, based on the smallest differences in RA content and hardness between the compared areas. In contrast, for the 750 °C sample, martensitic transformation and precipitates were detected by SEM. The martensitic transformation already has presumably occurred during cooling from intercritical annealing.

In the metallographic investigations, it was found that transformation of austenite may also occur during grinding and polishing steps. With decreasing stability of austenite because of the lack of C and Mn and effective austenite, the fraction size of polished samples was relatively low compared to etched samples used for XRD-Investigations. This must be taken into account and can explain, e.g., the lower RA content of 750 °C compared to the as-built state.

LPBF samples show expected mechanical behavior depending on annealing temperature and time compared to conventional material. However, the potential of achievable mechanical properties of medium manganese steels has not yet been fully exploited. Mn content of 4 wt.% is set in the lower range of typical medium manganese alloys (3%–12% Mn), higher contents offering higher strength and are currently being investigated. Additionally, mechanical properties were influenced by the remaining process defects, not fine pores, which cannot be completely avoided due to manganese burn-off, but larger bonding errors by melt spatters, resulting in high standard deviations in elongations at fracture, which must be further reduced to improve process stability.

Conventional medium manganese steel typically appears in the form of cold or hot rolled sheet metal. The thermo-mechanical rolling processes result in an ultra-fine grain microstructure and a high dislocation density. The characteristics of the LPBF process, especially the high cooling rates, however, lead to similar effects. Therefore, the process could potentially enable the manufacturing of solid components beyond sheet metals with similar properties and could open up new fields of application for this material.

## 5. Conclusions

The described process route has already demonstrated high potential for a transfer of the favourable mechanical properties of sheet metal medium manganese samples onto solid parts. Further investigations concerning the stability of the LPBF process and the interrelation of dislocation recovery during annealing and austenite stability are promising to achieve samples of high quality and excellent mechanical properties.

The following conclusions can be drawn from the study presented above:

- The composed medium manganese steel alloy was successfully gas atomized with a high powder quality. Burn-off of manganese during atomization and LPBF needs to be taken into account (21.4% in total).
- The powder could be processed by LPBF, reaching densities up to 99.9%. However, melt spatter occurred and led to bonding errors, resulting in high standard deviations of mechanical properties, especially in elongation. The effect of Si and Al on process instability needs to be further investigated.
- As-built microstructure is suitable to activate transformation induced plasticity (TRIP) in the LPBF-samples by intercrititical annealing, and mechanical behaviour was similar to conventionally produced medium manganese steel processed at comparable annealing temperature and duration.
- Austenite transformation could be detected by XRD and EBSD. The results suggest that metallographic grinding may already cause a transformation, and therefore, ablation by an etchant is preferable if possible.

## 6. Patents

The content of this and following studies has been applied as a patent at the German Patent and Trademark Office.

## Figures and Tables

**Table 1:** The parameters used for atomization process.

Parameter	Symbol	Unit	Value
Gas	—	—	Argon
Gas temperature	T_G_	°C	23
Gas pressure	P_G_	bar	12
Gas mass flow rate	${\;\dot{M}}_{G}$	kg h^−1^	583
Melt nozzle diameter	d_m_	Mm	2.5
Melt temperature	T_m_	°C	1715
Mean melt flow rate	${\dot{M}}_{m}$	kg h^−1^	265
Gas-to-melt ratio	GMR	—	2.2

**Table 2:** The chemical composition of the raw material used for the gas atomization, the produced powder and samples fabricated by LPBF in wt.%.

	Fe	C	Mn	Si	Al
Raw material	bal.	0.20	5.00	0.50	2.00
Powder	bal.	0.21	4.61	0.50	1.89
LPBF-sample	bal.	0.23	3.93	0.51	2.01

## Data Availability

The data presented in this study are available on request from the corresponding author after obtaining permission of authorized person.

## Appendix A

**Table A1:** The parameters used for modeling SFE: Grain Size D, Gibbs free energy Δ*Gγ*→*ε* for each element upon the phase transformation of austenite to martensite, interaction parameters *Ωγ*→*ε*, interfacial energy $\sigma^{\gamma /\varepsilon }$ and temperature T.

Parameter	Value	Unit	Reference
D	2	µm	estimated
$∆G_{Fe}^{\gamma \to \varepsilon }$	−1828.4 + 4.686 × T	J·mol^−1^	[49]
$∆G_{Mn}^{\gamma \to \varepsilon }$	3970–1.6667 × T	J·mol^−1^	[50]
$∆G_{Al}^{\gamma \to \varepsilon }$	5481.04–1.79912 × T	J·mol^−1^	[49]
$∆G_{Si}^{\gamma \to \varepsilon }$	−560 + 8 × T	J·mol^−1^	[51]
$∆G_{C}^{\gamma \to \varepsilon }$	−22,166	J·mol^−1^	[23]
$∆\Omega_{FeMn}^{\gamma \to \varepsilon }$	−9135.5 + 15,282.1 × XMn	J·mol^−1^	[52]
$∆\Omega_{FeAl}^{\gamma \to \varepsilon }$	3326.28	J·mol^−1^	[43]
$∆\Omega_{FeSi}^{\gamma \to \varepsilon }$	1780	J·mol^−1^	[53]
$∆\Omega_{FeC}^{\gamma \to \varepsilon }$	42,500	J·mol^−1^	[54]
ρ	2.966 × 10^−5^	mol·m^−2^	[55]
$\sigma^{\gamma /\varepsilon }$	10	mJ·m^−2^	estimated
T	293	K	
